# The Profession and the Voluntary Hospitals

**Published:** 1907-05-11

**Authors:** 


### THE PROFESSION AND THE VOLUNTARY HOSPITALS.

### PATIENTS PRACTITIONERS SHOULD SEND TO A HOSPITAL.

In introducing a discussion at a recent meeting of the Glasgow Southern Medical Society on the subject "When is a medical man justified in sending a patient to hospital?" Dr. John Steward made some pertinent remarks on the factors which should guide the practitioner in "sending in" a patient.

#### The Case for the Authorities.

"All will agree that the hospital is a charitable institution primarily intended for those unable to pay for medical attendance, medicine, and nursing. If hospital authorities acted up to this charitable intention, as they ought to do, they would have good grounds for objecting to practitioners sending in patients whose circumstances do not call for charitable consideration. As it is, hospitals have opened their doors so widely that practically anyone who chooses may enter therein. Statistics show that one-sixth of the population of Glasgow is annually in receipt of some form or other of medical charity, and it may safely be inferred that many obtain admittance to the infirmaries though quite able to provide for themselves. There is no evidence that the authorities are taking sufficient steps to prevent what we consider to be an abuse of medical charity, and they have no right, therefore, to criticise adversely the action of a practitioner, who endeavours to obtain for his patient the benefits of a hospital. A medical man is never 'not justified' in sending in a patient who is critically ill, no matter what the latter's financial position may be, and it is unlikely that the hospital authorities will take the practitioner to task for doing so. But while the authorities are not justified in allowing the charity to be abused, a medical man can never be fully excused for ignoring the primary object upon which hospitals base their claim for the consideration of a charitably disposed community."

#### The Patient's Position.

"A doctor's first duty is to his patient. When gravely ill, the latter is entirely dependent on those around him for nursing care, and to know that he receives such care is the constant and anxious desire of his medical attendant. If the patient is not a dependent according to law, and if those in whose charge he is are not legally bound to give him the necessary care and attention, or are not able or willing to do so, or if assistance be offered in a grudging and perfunctory manner, then the question to be considered is: 'Can the patient himself pay for such care without overtaxing his financial resources?' Unless he can afford to do so, the doctor is justified in sending him to the hospital, and it is the doctor's duty to do so."

"Again, if the patient is a lawful dependent, and those in charge of him are legally bound to provide for him, but are not able, or, being able, are decidedly unwilling or refuse to do so, then the medical man is justified in sending him in. In the case of domestic servants, for example, where the employers refuse reasonable medical aid and nursing care, the medical man is not called upon to waste time and attention in a vain attempt to bring the employers to a sense of their responsibilities. They should certainly not be allowed to escape, but the doctor's first care should be for the welfare of the patient, and the onus of compelling those who are financially able to fulfil their legal obligations should rest with the hospital authorities, who should have power to enforce their authority."

"Then, again, we are justified in sending in cases where those in charge of the patient who is dangerously ill have taxed their energies and resources to the uttermost, and, though still perfectly willing to do all that in their power lies, are obviously unable to keep up unflaggingly the care and attention needful for the patient's safety. We may formulate, as a general principle, the rule that when a patient is seriously ill, and where those on whom he is dependent are unable or unwilling to provide for him, the medical man is justified in sending him in. In such a case we may rightly ignore all considerations except the patient's interest."

#### Ourselves and our Neighbours.

"The great majority of our cases may be divided into two classes. First, those with which we feel ourselves incompetent to deal. Secondly, those which are not progressing under our care, and in which we are not sure of our ground. For these reasons, however, no man is justified in taking advantage of the benefits of a hospital. If medical men would seek the assistance of their neighbours more freely they would soon find less difficulty in dealing with many minor cases which are so frequently sent to hospital and dispensaries. If they would not hesitate to seek the help of a consultant their uncertainties about a case would be dispelled, and they would with more assurance, an easier mind, be able to go on with it. If the case be one which cannot be dealt with at home, the patient is even one step higher in the social scale than the working class, then the hospital should not be suggested until the question of expense has been discussed with the consultant, and the matter laid fairly before the patient's friends. Until a man has given some consideration to the financial aspect of the question from the point of view of the profession generally, he is not justified in ordering removal to a hospital. Remember that everyone sent to the hospital may lead half a dozen others to it. Therefore it is your duty, as a medical practitioner, to dissuade patients living in a well-to-do residential district from going to the infirmary, as others from the same district will have no hesitation in following their example; and the idea of the hospital as a charitable institution will become weaker and weaker in the mind of the public. Rather have the expenses modified as much as possible. Indeed, if necessary, sacrifice your own financial interests entirely, in the case of a regular patient, if by so doing he can be kept from the hospital. It will repay you in the end to do so. Of course, I do not suggest straining the resources of anyone too far. It is your moral duty to protect your patients' interest in every way."

